# Metabarcoding of fungal assemblages in *Vaccinium myrtillus* endosphere suggests colonization of above-ground organs by some ericoid mycorrhizal and DSE fungi

**DOI:** 10.1038/s41598-022-15154-1

**Published:** 2022-06-30

**Authors:** Stefania Daghino, Elena Martino, Samuele Voyron, Silvia Perotto

**Affiliations:** 1 Institute for Sustainable Plant Protection, CNR, Strada delle Cacce 73, 10135, Torino and V. le Mattioli 25, 10125 Torino, Italy; 2 Department of Life Sciences and Systems Biology, University of Torino, V. le Mattioli 25, 10125 Torino, Italy

**Keywords:** Metagenomics, Symbiosis, Microbiome, Plant symbiosis

## Abstract

Plants harbor in their external surfaces and internal tissues a highly diverse and finely structured microbial assembly, the microbiota. Each plant compartment usually represents a unique ecological niche hosting a distinct microbial community and niche differentiation, which may mirror distinct functions of a specialized microbiota, has been mainly investigated for bacteria. Far less is known for the fungal components of the plant-associated microbiota. Here, we applied a metabarcoding approach to describe the fungal assemblages in different organs of *Vaccinium myrtillus* plants (Ericaceae) collected in a subalpine meadow in North-West Italy, and identified specific taxa enriched in internal tissues of roots, stems, leaves and flowers. We also traced the distribution of some important fungi commonly associated with plants of the family Ericaceae, namely the ericoid mycorrhizal (ErM) fungi and the dark septate endophytes (DSE), both playing important roles in plant growth and health. Operational taxonomic units attributed to established ErM fungal species in the genus *Hyaloscypha* and to DSE species in the *Phialocephala-Acephala applanata* complex (PAC) were found in all the plant organs. Mycorrhizal fungi are thought to be strictly associated with the plant roots, and this first observation of ErM fungi in the above-ground organs of the host plant may be explained by the evolutionary closeness of ErM fungi in the genus *Hyaloscypha* with non mycorrhizal fungal endophytes. This is also witnessed by the closer similarities of the ErM fungal genomes with the genomes of plant endophytes than with those of other mycorrhizal fungi, such as arbuscular or ectomycorrhizal fungi.

## Introduction

Plants live closely associated with complex microbial assemblages, or microbiota, that colonize plant surfaces as well as internal tissues and include nematodes, fungi, unicellular eukaryotes, bacteria, archaea and their infecting viruses^1^. The plant-associated microbiota can play a key role for plant health, development and productivity, and a plant with its associated microbiota, the “holobiont”, can be considered as a single entity that evolves in the environment and time, thanks to the co-evolution of the single components interacting with each other^2^.

Components of the microbiota inhabiting the plant internal tissues for at least part of their lifetime without causing disease are termed endophytes^3^, although broader definitions of “endophyte” based on habitat only and including all microbes inhabiting plant tissues have been proposed^1^.

Fungi are functionally dominant and ubiquitous in the plant microbiota, and fungal endophytes have been found in all plant species studied to date. Rodriguez and colleagues^4^ classified endophytic fungi according to their colonization pattern (i.e., systemic or organ-specific), transmission (i.e., vertical or horizontal) and phylogeny (i.e., clavicipitaceous or non-clavicipitaceous). Endophytic fungi are often considered to be beneficial to their host plants because they can provide resistance against pathogens and insect herbivores^5^. They can also confer stress tolerance, such as salt and heat tolerance^6^ and promote plant root formation and shoot growth^7^. On the other hand, asymptomatic endophytic fungi could become pathogens under stressful conditions, or they could have long latent periods^8^.

Mycorrhizal fungi also colonize the internal root tissues, where they form intimate symbioses whose morphological and functional features depend on the plant and the fungal taxonomic position^9^. It has been debated if mycorrhizal fungi are part of the endophytic fungal community, or whether they belong to a different guild^1,10,11^. Although broad definitions of endophytes usually include mycorrhizal fungi, many authors (including ourselves) consider mycorrhizal fungi as distinct from endophytes, based on different criteria. For example, Rodriguez^4^ excluded mycorrhizal fungi from the term endophyte because, in addition to internal root tissues, many mycorrhizal fungi extensively grow outside the rhizosphere into the soil. The formation of specialized fungal structures within the plant tissues also excluded mycorrhizal fungi from the definition of endophytes given by Wilson^10^ “fungi or bacteria which, for all or part of their life cycle, invade the tissues of living plants and cause unapparent and asymptomatic infections entirely within plant tissues but cause no symptoms of disease”. To avoid confusion in the use of the term endophyte, given the different interpretations, in this paper we will refer to fungi colonizing internal plant tissues as “endospheric” fungi, a general term already used by other authors^12–14^.

Irrespective of the definition and spectrum, many fungi colonizing internal plant tissues seem to be unequally distributed in the plant organs. For example, according to the classification by Rodriguez and colleagues^4^, Class IV endophytes, namely the dark septate endophytic (DSE) fungi, are restricted to the roots, which they colonize extensively in a wide range of host plants. A similar unequal distribution pattern has been found for bacteria associated with plant surfaces and internal plant tissues, both above- and below-ground, where it has been suggested that these plant compartments may represent a major selective force that shapes the composition of plant-associated microbiota^2^. When compared to the communities of bacterial endophytes, variation in the fungal assemblages within the different plant niches is still poorly known, and although several studies have focused on the plant-soil interface, less is known about the patterns of fungal diversity in the different plant compartments^12^. It is for example unclear whether fungal distribution in the plant mainly depends on the taxonomic position of the fungus, or on specific constraints posed by the different plant compartments.

To increase our knowledge on the fungal assemblages associated with above- and below-ground plant tissues, and to address specific questions on the distribution of some key components of plant-associated fungi, we have investigated the diversity of the fungal assemblages in the endosphere of *Vaccinium myrtillus* (Ericaceae).

Plants belonging to the Ericaceae family, encompassing 4426 species and around 129 genera^15^, represent important components of the heathland flora and some open forest communities worldwide. These geographically and climatically disparate habitats rely on soils that are usually very poor in mineral nutrients but can be enriched in aromatic compounds and potentially toxic metals, made readily available by the generally low pH^16^. About 30 genera in the Ericaceae family^17^ are characterized by a peculiar endomycorrhizal symbiosis, namely the ericoid mycorrhiza (ErM), and the adaptation of these plants to their stressful habitats has been also attributed to the ability of their associated ErM fungi to increase the host plant fitness^18^. The role of non-mycorrhizal endospheric fungi in the adaptation of Ericaceae to stressful conditions is far less understood, although DSE fungi^4^ are commonly isolated from the roots of ericaceous plants^19–23^ and they enhance plant performance when inoculated under controlled conditions^24^. Furthermore, some DSE fungi may form intracellular structures morphologically resembling the ErM symbiosis^25^.

Beside playing a crucial ecological role in heathland habitats, some genera of Ericaceae have a commercial interest as agronomic cultures in the flower and horticultural industry, both as food and nutraceutical sources, thanks to their high content in secondary metabolites^26^. Ericoid mycorrhizal fungi have been demonstrated to influence not only plant fitness in the field, but also some plant phenotypic traits, such as flower size and fruit number and quality^27^.

Fungi associated with ericaceous plants have been mainly investigated by culture-dependent methods, most studies being focused on the isolation and identification of ErM fungi. Controversy surrounded the earliest attempts to identify the fungi involved in the formation of ErM. The fungus *Phoma radices-callunae*, isolated by Rayner^28^ from roots as well as from shoots and floral organs of ericaceous plants, was claimed to be a mycorrhizal as well as a systemic symbiont, vertically transmitted through the seed coat. However, *P. radices-callunae* did not form hyphal coils within the root epidermal cells, typical of ErM, and is now described as a Class 2 endophyte colonizing all plant parts^4^. This fungus has been recently reclassified as *Didymella anserina*^29^.

True mycorrhizal coil-forming ErM fungi are mainly ascomycetes in the class Leotiomycetes, the most recent phylogenetic revision of ErM fungi placing the dominant fungal symbionts in the genus *Hyaloscypha*^30^. The first fungal species experimentally confirmed as ErMF^31^ is now recognized as *Hyaloscypha hepaticicola* (was previously classified as *Pezoloma ericae, Rhizoscyphus ericae, Hymenoscyphus ericae* and *Pezizella ericae*^32^). Many sterile isolates from ericaceous roots, later classified by molecular methods and placed in the species complex “*Hymenoscyphus ericae* aggregate” (REA^33^), are also congeneric with *Hyaloscypha*^30^, including the recently described ErM fungal species *H. gryndleri*^34^. In particular, confirmed ErM fungal species in the genus *Meliniomyces* are now reclassified as *Hyaloscypha variabilis* and *H. bicolor*^35–37^. The REA also include *Hyaloscypha finlandica* (formerly *Cadophora finlandica*), a species reported to form ectomycorrhiza with conifers and ErM with ericaceous plants^38,39^, as well as other mycorrhizal and non-mycorrhizal fungi^40^. Outside the genus *Hyaloscypha*, fungi identified as *Oidiodendron maius* have been often isolated from mycorrhizal roots of ericaceous plants and shown to form typical hyphal coils^41^.

Other ascomycetes have been sporadically reported to form hyphal coils in the roots of ericaceous plants in vitro and are considered as putative ErM fungi, although the mycorrhizal function of some of these associations is still under debate (see^40^ and references therein). They include some Helotiales that can form functional ErM, isolates in the genus *Leohumicola*, *Acremonium strictum*, *Geomyces pannorum,* some DSE fungi of the *Phialocephala*-*Acephala applanata* complex (PAC^25^), isolates with affinities to the genera *Capronia*, *Cadophora*, *Cryptosporiopsis* and *Lachnum*, fungi belonging to an unnamed lineage in the Chaetothyriomycetidae^42^.

Basidiomycetes in the genus *Serendipita* (Sebacinales, Agaricomycetes) are also common inhabitants of ericaceous roots, where they form typical hyphal coils^43^. A member of the *Kurtia argillacea* species complex (Agaricomycetes) identified by Vohník and colleagues^44^ from *Vaccinium* spp., has been considered as a putative ErM fungus because it forms intracellular structures with a unique morphology, described as a “sheathed-ericoid” mycorrhiza.

Many recent studies^20,21,23,45–49^ have investigated the root-associated fungal assemblages of ericaceous plants by culture-dependent and independent methods, whereas few investigations have focused on the diversity of endospheric fungi in above-ground organs. Petrini^50^ isolated fungi from the leaves of different ericaceous species, including *V. myrtillus*, Li and colleagues^51^ analyzed the diversity of fungal assemblages inside fruits, leaves and branches of *V. dunalianum* var. *urophyllum* (known as South China blueberry), whereas Koudelkova and colleagues^52^ isolated endospheric fungi from *Rhododendron tomentosum* leaves. Thus, information about fungal diversity in plant compartments different from the roots is limited in the Ericaceae. Here, we investigated by metabarcoding the fungal diversity of internal tissues of both below- and above-ground organs of field collected plants of *V. myrtillus* (European blueberry), with the aim to verify if distinct plant compartments (i.e., roots, stems, leaves and flowers) harbor similar or significantly different assemblages of endospheric fungi.

In addition, we investigated the distribution of some established ErM and DSE fungi in the host plant. Ericoid mycorrhizal fungi can be found as non-mycorrhizal endospheric fungi in non-ericaceous hosts^37^ and recent data indicate that some genomic features of sequenced ErM fungi^53^ and the DSE fungus *Phialocephala* *subalpina*^54^ are similar to those of other fungal endophytes, with an expansion of the repertoire of Carbohydrate Active enZymes (CAZymes) and an unusually high number of genes coding for polyketide synthases involved in the biosynthesis of bio-active secondary metabolites^53^. Thus, we hypothesize that some root-associated fungi may be more versatile in their trophic strategies and colonization potential than traditionally thought.

## Results

### Fungal diversity associated with the different plant organs

The fungal assemblages associated with the four organs of *V. myrtillus* (i.e., roots, stems, leaves and flowers) were investigated by high-throughput sequencing of the fungal ITS2 region. After removal of low-quality reads, we obtained in total 2,863,742 high quality reads (maximum counts per sample: 188,914; minimum counts per sample: 93,654) corresponding to 1,621 Operational Taxonomic Units (OTUs; 97% similarity), among which 1186 had ≥ 2 counts. After discarding OTUs with low counts (less than 10 reads) and low standard deviation (see Material and methods), 749 OTUs were retained.

The alpha diversity of fungal assemblages in the four plant organs was assessed by calculating the Chao1 and Shannon indices. The Chao1 index, which estimates richness based on taxa abundance, showed no significant differences among organs (Kruskal–Wallis p-val = 0.08; Supplementary Fig. S1), while the Shannon index, that considers both richness and evenness (abundance distribution across species), revealed a significant difference among organs (Kruskal–Wallis p-val = 0.047; Supplementary Fig. S1), with the highest fungal diversity in leaves. No significant differences were found in the alpha-diversity values of the different samples of each organ.

**Figure 1:**
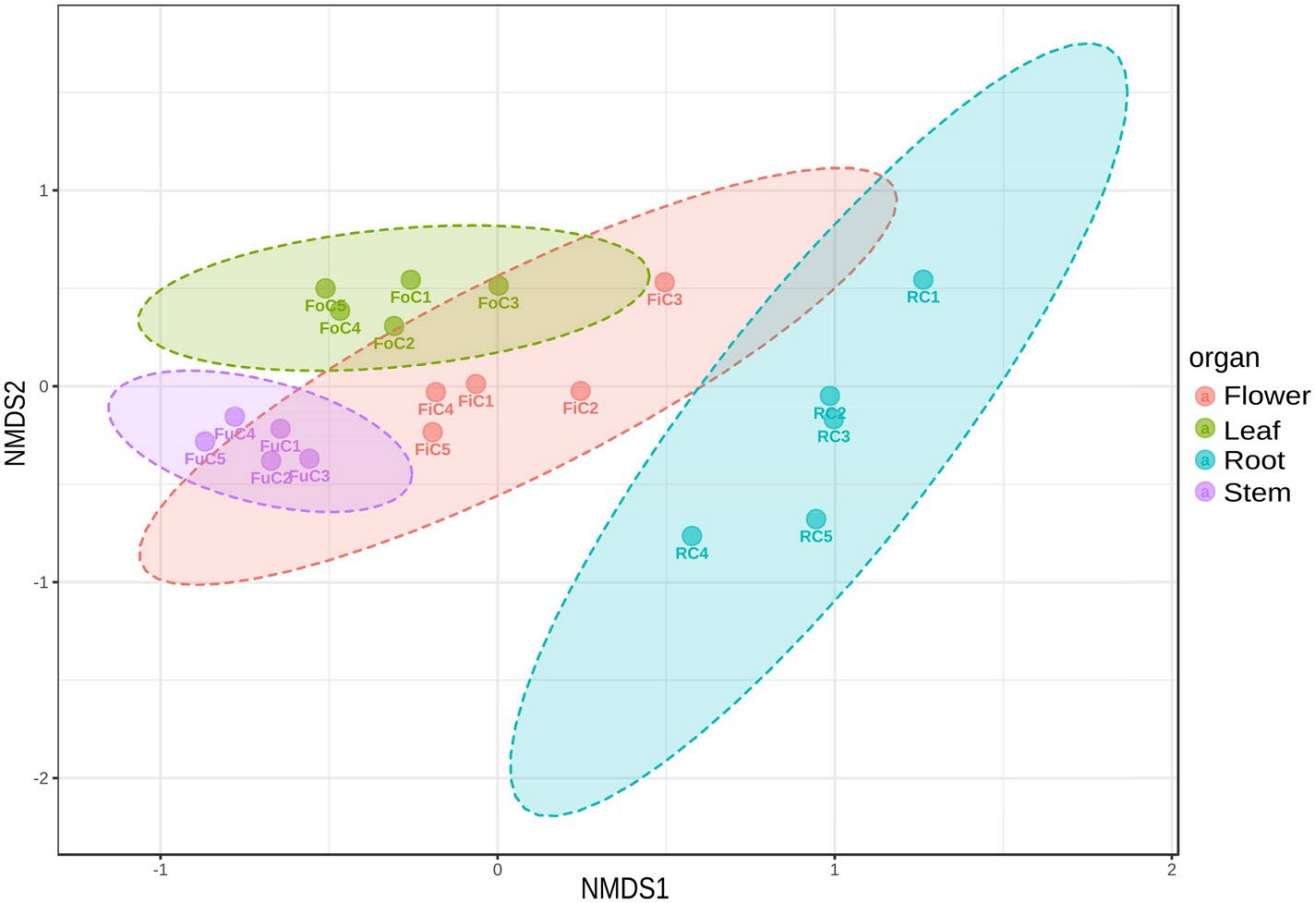
Beta-diversity of the fungal assemblages associated with the different plant organs. The beta-diversity among the different organs was estimated by a NMDS analysis based on Bray–Curtis dissimilarities, with the following parameters: taxonomic level: feature, statistical method: PERMANOVA, experimental factor: organ. Fi = Flowers, Fo = Leaves, Fu = Stems, R = Roots. C1-C5: samples.

Beta-diversity was estimated by NMDS based on Bray–Curtis dissimilarities and showed that the fungal assemblages of stems, leaves and flowers were partially overlapping, whereas the fungal assemblage in the root samples clustered separately in the ordination space (Permanova F-val = 4.35, R^2^ = 0.449, p-val < 0.001, NMDS stress = 0.133; Fig. 1).

**Figure 2:**
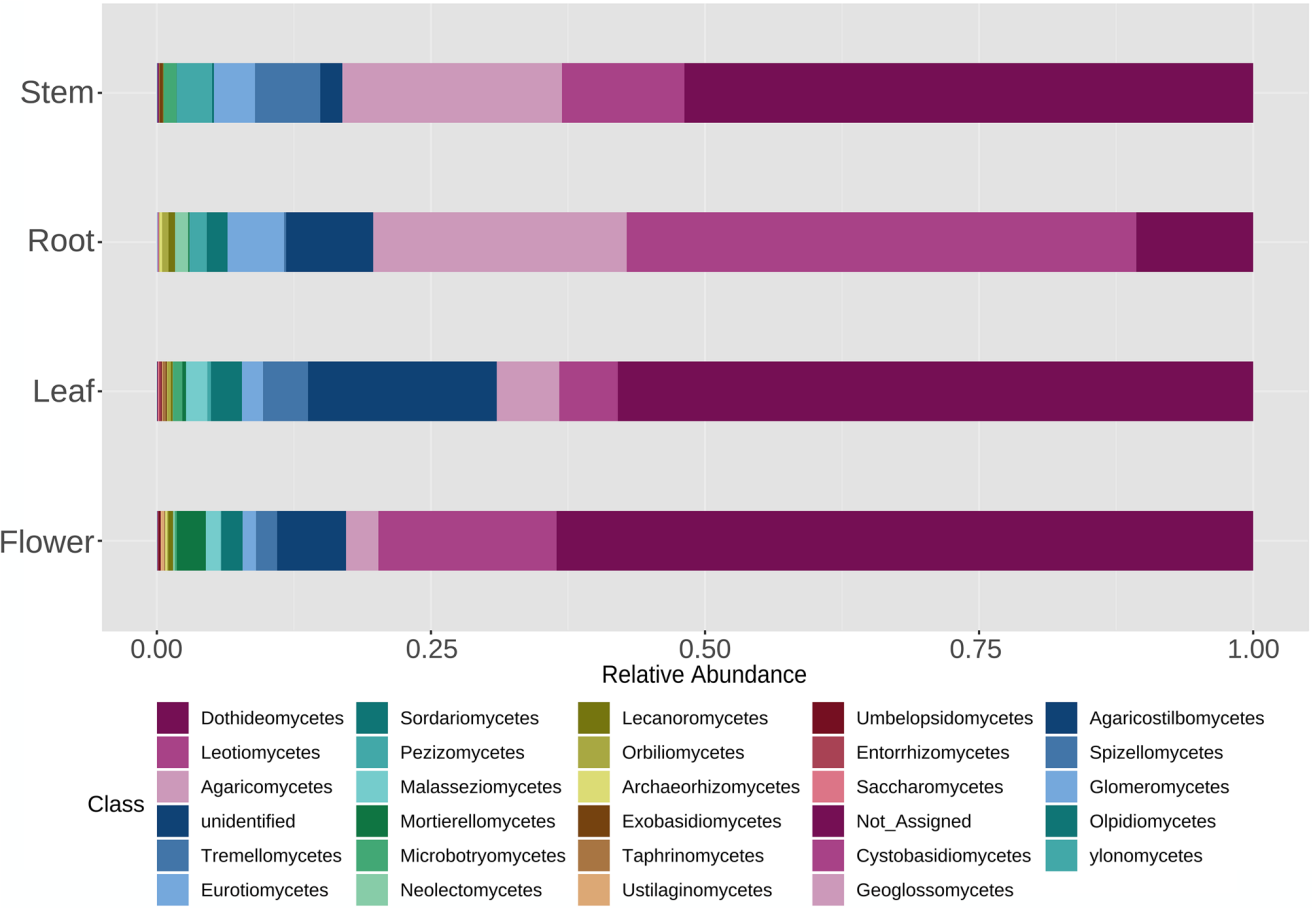
Description of the fungal diversity associated with different plant organs: relative abundance of fungal classes.

At the phylum level (Supplementary Fig. S2), the fungal population associated with *V. myrtillus* was dominated by Ascomycota (representing from 69 to 84% of the taxa in the different plant compartments), followed by Basidiomycota (from 7% of the taxa in the flowers up to 27% in the stems) and by all the other phyla with percentages below 1% (Glomeromycota, Mortierellomycota, Mucoromycota, Olpidiomycota). A small percentage of taxa (6%) corresponded to unidentified and not assigned phyla. The phylum Basidiomycota was significantly more abundant in stems than in flowers and leaves, while Chytridiomycota were more abundant in stems than in leaves (Supplementary Fig. S2). At the class level (Fig. 2), Dothideomycetes was the most abundant (46% on average in the different compartments), followed by Leotiomycetes (20%), Agaricomycetes (13%) and by the other classes with percentages below 1%. Overall, 8% of the total taxa corresponded to unidentified and not assigned classes. Among all classes, only eight showed significant differences in their abundance across the different organs and are shown in the Supplementary Fig. S3. In particular, the classes Dothideomycetes and Tremellomycetes were significantly less abundant in roots than in all the other organs, whereas Leotiomycetes and Neolectomycetes were significantly more abundant in roots than in all the other organs. At the genus level (Supplementary Fig. S4), the unidentified taxa in the different plant organs represented on average the 64%, and the most abundant identified genus was *Phialocephala* (overall 3.6%). According to the identification based on the UNITE database, among the genera including known ErMF we found *Hyaloscypha, Pezoloma and Meliniomyces* (Supplementary Table S1)*,* representing all together from 0.3% of the taxa in the leaves to 5% of the taxa in the roots. Since these genera have been recently taxonomically revised^30,34^, we performed a phylogenetic analysis including such OTUs (Supplementary Fig. S5) and attributed them to different species in the genus *Hyaloscypha* (namely *H. finlandica*, *H. gryndleri*, *H. hepaticicola*, *H. vraolstadiae* and several *Hyaloscypha* spp.). We referred to this attribution in the subsequent analyses. The genus *Oidiodendron* (0.006% of the taxa) was also identified in the database, represented by the ErM species *O. maius* and by the non-ErM species *O. chlamydosporicum, O. griseum* and some *Oidiodendron* spp. (Supplementary Fig. S6). Among the genera featuring putative ErM fungi, according to the literature^40^, were *Lachnum* (0.008%) and *Capronia* (0.04).

**Figure 3:**
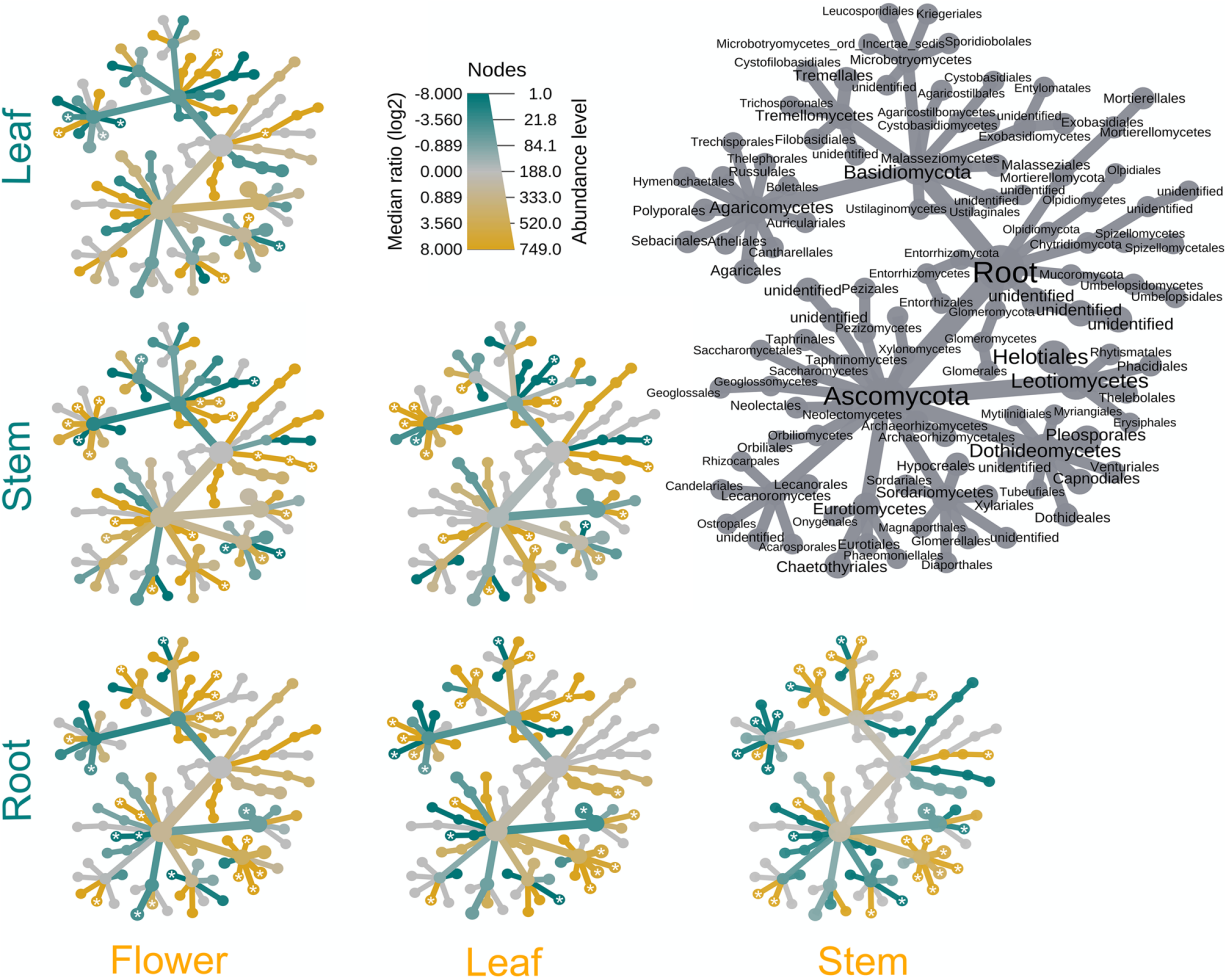
Heat tree matrix depicting the different taxa abundance among the plant organs, for all orders in the dataset. The size of the nodes in the gray cladogram (right) represents the number of OTUs identified at that taxonomic level. The small cladograms show the pairwise comparisons among the organs: a yellow node indicates a higher abundance of the taxon in the organ indicated in yellow than in the organ indicated in green. A green node indicates the opposite. Taxa identified as differently represented, statistically supported by the Wilcoxon test (p < 0.05), are tagged with a white asterisk.

Organ-wise comparisons of relative abundance of fungal orders (Fig. 3) showed the highest number of significantly different taxa when roots were compared with all the other organs. In particular, the orders Helotiales and Leucosporidiales were always more abundant in roots than in the other organs, while Dothideales and Capnodiales, both in the class Dothideomycetes, were less abundant in roots. Sebacinales were more abundant in roots than in leaves and stems. Atheliales were more abundant in stems than in the other organs, while Polyporales were more abundant in leaves and Capnodiales were more abundant in flowers than in stems.

The LefSe score (Linear discriminant analysis Effect Size^44^) was used to estimate differences in the relative taxa abundance among organs at the class, order and genus level (Supplementary Fig. S7). Few taxa were identified that could be considered as markers of the different organs. Roots were enriched (LogLDA > 3) in the classes Leotiomycetes (with the order Helotiales and the genus *Hyaloscypha*) and Agaricomycetes (with the orders Agaricales*,* Thelephorales and Sebacinales, with the genus *Serendipita*). Stems were enriched in the class Tremellomycetes (with the order Tremellales) and in the orders Pleosporales, Atheliales and Dothideales. Leaves were enriched in the genera *Perusta*, *Didymella* and *Pseudopithomyces*, while flowers were enriched in the class Dothideomycetes (with the order Capnodiales) and in the genus *Botrytis*. Principal Component Analysis (PCA) of OTUs distribution (Supplementary Fig. S7) showed that the differences between organs were driven by few single OTUs. In particular, roots were characterized by OTUs 39, 716, 719, 736 (respectively identified as *Pseudotomentella* sp., PAC*, Lachnellula pulverulenta* and *Dothideomycetes* sp.), flowers by OTUs 98, 591, 621 (all assigned to Cladosporiaceae), stems by OTUs 457, 2270, 704, 411 (the first being assigned to *Atheliales* sp*.*, the second and third to Melanommataceae in the Dothideomycetes, the last being assigned to Dothideales).

**Figure 4:**
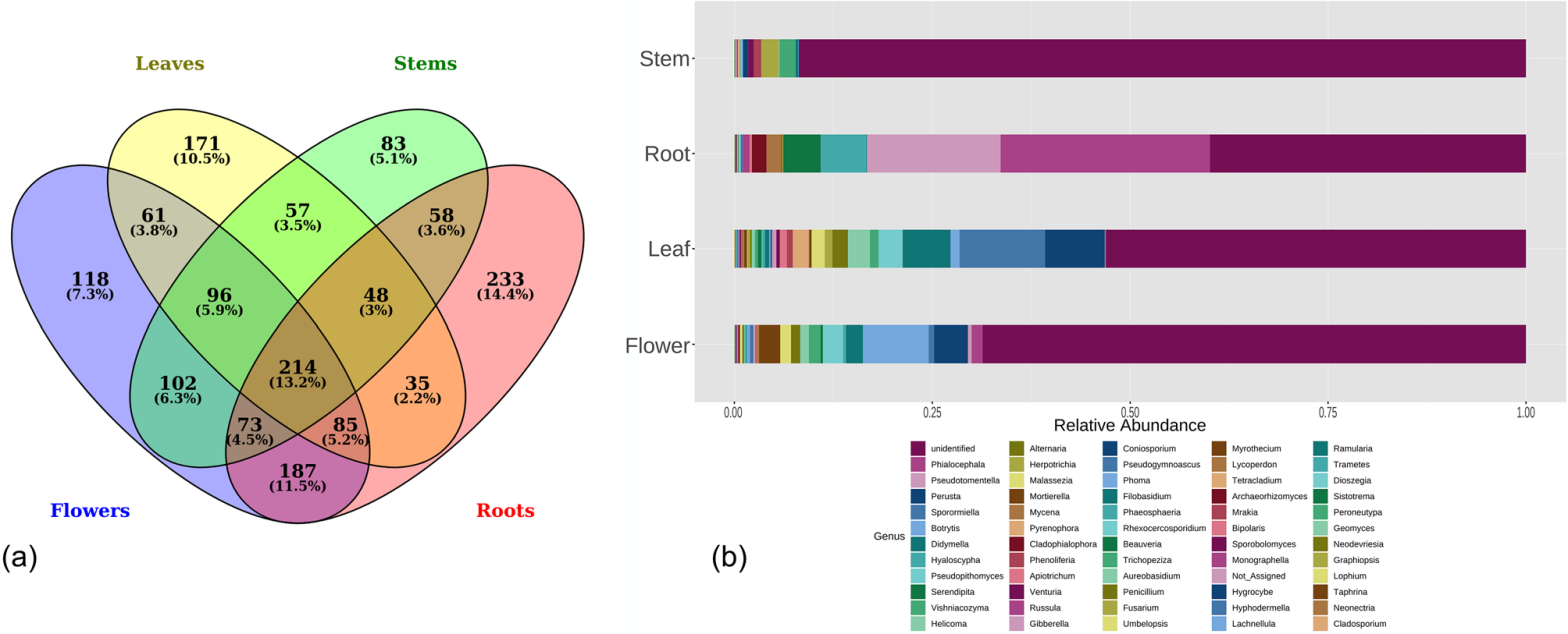
OTU distribution in the different samples and fungal genera in the core OTUs. (**a**) Venn diagram of the OTUs from the different plant organs; (**b**) relative abundance of the genera within the 214 core OTUs.

A core of 214 OTUs was found in all four plant organs (Fig. 4). Among them, the most abundant genera were *Phialocephala* and *Pseudotomentella* (syn. Polyozellus*)*, mainly detected in roots, *Perusta* and *Sporormiella*, mainly detected in leaves, and *Botrytis,* mainly detected in flowers. Interestingly, among the core OTUs were genera including known or putative ErM fungal species, such as *Hyaloscypha* and *Serendipita*, as well as genera including DSE fungi, such as *Phialocephala,* or ectomycorrhizal fungi, such as *Russula* and *Pseudotomentella* (Fig. 4).

**Figure 5:**
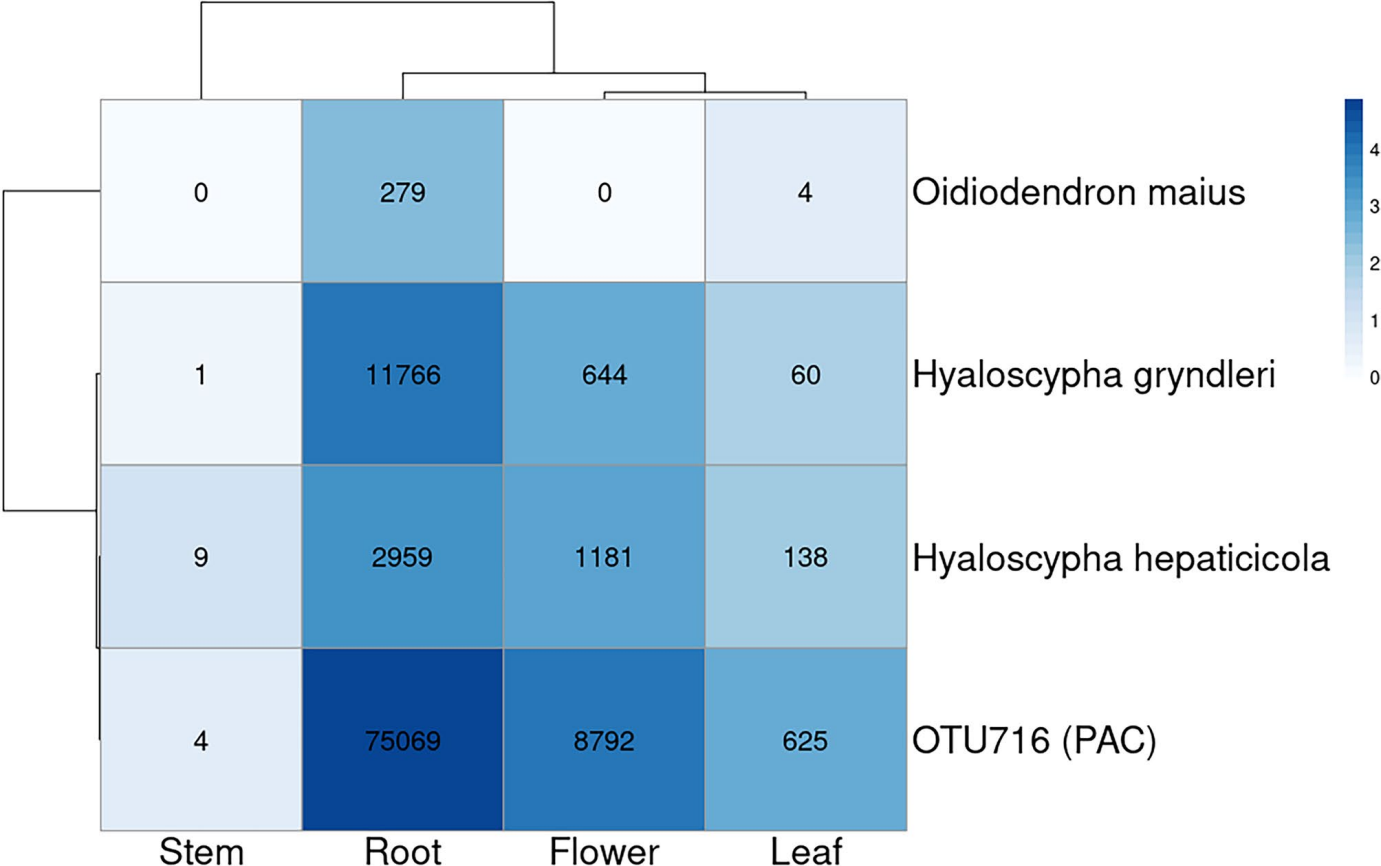
Distribution of reads from the mycorrhizal species *O. maius*, *H. gryndleri* and *H. hepaticicola*, and the core OTU716 assigned to the DSE *P. fortinii* (PAC) in the *V. myrtillus* organs (double clustering based on average linkage algorithm and Pearson correlation). Numbers in the matrix represent the absolute reads supporting the OTUs. The color key represents the log scale of the number of reads.

In our database, the genus *Hyaloscypha* included ErM fungi (i.e., *H. gryndleri* and *H. hepaticicola*) as well as non ErM species (i.e., the ectomycorrhizal *H. finlandica*). Similarly, the genus *Phialocephala* included DSE (i.e., the OTUs in the PAC) and non DSE (e.g., *P. fluminis*) species. Therefore, we further investigated the distribution of established ErM and DSE fungal species in the different plant organs by double-clustering analysis (Fig. 5). Although it was not a core OTU, we also included in the analysis the ErM fungal species *O. maius*. The result of the double-clustering analysis showed that the two *Hyaloscypha* ErM fungal species were not restricted to the root samples, as they were also detected in leaves and flowers and, although with a low number of reads, in stems. This pattern of colonization was much more similar to the DSE fungus *P. fortinii* than to the other ErM fungus *O. maius*, whose distribution was limited to roots and leaves. However, it should be noted that the overall number of reads was low for *O. maius*.

## Discussion

The plant internal tissues represent a unique ecological niche where some distinctive fungal species may live. These plant-fungus associations play an important role in the adaptation to the environment of both plants and fungi, together with the other organisms that constitute the holobiont.

Here, we have used a culture-independent approach to investigate the fungal assemblages inhabiting different organs of *V. myrtillus* (Ericaceae) plants collected in an alpine habitat. At a coarse taxonomic level, the endospheric fungal assemblage was dominated by Ascomycetes, followed by Basidiomycetes. This is in agreement with previous studies on the root-associated fungi of Ericaceae^20,21,23,45–47^ but in contrast with the report of Trivedi and colleagues^2^ that, based on the analysis of metabarcoding datasets from different angiosperms, stated that the endospheric fungal assemblages were dominated by Basidiomycetes.

Association of some fungal endophytes with specific host tissues has been observed in some plant species^55^. Similarly, in *V. myrtillus* we showed that the different organs shape the endospheric fungal assemblages, with some niche differentiation. The analysis of beta-diversity revealed that the root endosphere was colonized by distinct fungal assemblages, possibly because of the closeness and influence of the rhizopheric soil.

Alpha-diversity indices suggest a similar degree of fungal diversity within the *V. myrtillus* organs, except for the diversity associated with leaves, that was higher when evenness was taken into consideration by the Shannon index. This is in line with some previous reports^2,51^ and suggests that leaves can host a heterogeneous fungal assemblage, possibly because of the influence of fluctuating environmental factors.

Relative abundance of lower rank taxa revealed that the Helotiales were more abundant in roots than in the other organs, and the genus *Hyaloscypha,* comprising both ErM and non-ErM fungal species, could be considered as a biomarker of the root compartment. In addition, one of the OTUs that determined the divergence of the root compartments from the other plant compartments was assigned to *Phialocephala fortinii.* This species belongs to the group of the DSE fungi and forms with *A. applanata* the so-called *P. fortinii* s.l.—*A. applanata* species complex (PAC), often found associated with Ericaceae roots^19,23^. Sebacinales were also more abundant in roots than in leaves and stems, with *Serendipita* as a biomarker of the root compartment. Sebacinales have been already reported as common fungi in *Vaccinium* spp. roots^19,23,45^ and encompass ubiquitously distributed taxa found as symbionts in diverse mycorrhizal types, ranging from ectomycorrhiza to ericoid and orchid mycorrhiza, and as root endophytes. Species belonging to the Leucosporidiales, found to be more abundant in roots that in the other organs, have been already found in leaves and stems both in grasses and in woody plants^56,57^ but, to our knowledge, they have never been reported from roots.

Among the dominant genera in the roots, we also detected typically ectomycorrhizal fungi such as the genera *Russula, Polyozellus* (formerly *Pseudotomentella*), possibly originating from the neighbouring tree species, as well as the species *Hyaloscypha finlandica*. Ectomycorrhizal fungal genera (*Russula, Tomentella, Rhizopogon, Thelephora, Cenococcum*) were previously found in the roots of *V. carlesii* by Zhang and colleagues^45^, but it is unclear whether these fungi are occasional occupants of non-ectomycorrhizal roots, or whether they play some functional roles in the root endosphere of ericaceous plants. The association of *H. finlandica* with *V. myrtillus* also remains to be established, given the dual mycorrhizal behaviour of this species^30^.

In the stems, the analyses indicated the dominance of fungi in the order Pleosporales, Atheliales, Dothideales and Tremellales, as well as OTUs that found no reliable matching in the current databases. Capnodiales represented a hub taxon for the flowers and *Botrytis* genus within the Helotiales could be considered as a biomarker of this compartment. Detrimental species of *Botrytis* (e.g., *B. cinerea*) have been already detected in flowers and fruits of *Vaccinium* spp.^58^.

Although the results of metabarcoding indicate niche differentiation of the endospheric fungal assemblages in the plant organs, likely due to distinct microenvironment filtering and different microbial inoculum source, we also identified a core microbiota consisting of fungi that were ubiquitous in the plant endosphere. Members of the core microbiota that can influence the community structure through strong biotic interactions with the host or with other microbial species are defined as ‘hub microorganisms’^59^*.* Few dominating taxa in a single *V. myrtillus* organ turned out to be present, although with lower reads numbers, in other organs as well, being part of the host core microbiota. This was the case, for example, for the genera *Phialocephala*, *Athelia* and *Cladosporium, Alternaria* and *Dydimella*. The identification of *Phialocephala* in the core microbiota, represented by a single OTU assigned to the PAC, was interesting because DSE fungi belonging to this species complex are reported as root-specific endophytes^60^. Some OTUs (13 Ascomycota and 3 unidentified) were detected in all samples from all organs, suggesting that they might be associated with the understorey vegetation of the alpine field analyzed, but a more extensive analysis on different plant species from the same field site would be necessary to support this hypothesis.

Concerning the mycorrhizal components of the endospheric fungal assemblages, most established ErM fungal species in the ascomycetes were revealed in our metabarcoding experiments. A single OTU was identified as *O. maius*, the only species in the genus *Oidiodendron* known to form ErM*,* and it occurred almost exclusively in roots. However, the overall low number of reads suggests that this species was not abundant in the sampled area and makes it difficult to draw conclusions on its real distribution in the *V. myrtillus* organs. By contrast, well-established ErM fungal taxa in the *Hyaloscypha* genus, such as *H. hepaticicola* and *H. gryndleri*, each supported by thousands of reads, were part of the core endospheric fungal assemblage of *V. myrtillus*. Thus, these findings suggest that some fungi reported to be exclusively associated with the root endosphere may also colonize other plant organs. Although these results require further confirmation, such as the isolation of ErM fungi from field-collected *V. myrtillus* stems/leaves and plant inoculation *in vitro*^61^, they raise intriguing issues on the life strategies of these fungi.

Taxonomically, ErM and DSE fungi are placed within the class Helotiales, which includes many endophytic fungi^40^. In particular, the best characterized ErM fungal species belong to the genera *Oidiodendron, Hyaloscypha* and *Serendipita*, which also comprise known saprobes and endophytes. The genomic features of ErM and DSE fungi^53,54,62^, share many similarities with those of fungal endophytes colonizing aerial plant parts, such as *Sarocladium brachiariae* in the Sordariomycetes^63^. Common features are the large number of genes involved in plant cell wall degradation and biosynthesis of secondary metabolites, such as polyketides. Although these genomic features may suggest, at least for some ErM and DSE fungi, a systemic colonization of the aerial plant endosphere, the number of reads was very uneven in the different plant organs. In fact, it was quite surprising to find, for ErM and DSE fungi as well as for many core fungal OTUs, a higher number of reads in the flowers than in the leaves or stems of *V. myrtillus*. A recent study on bacteria colonizing the internal tissues of some orchid species has identified in the orchid flowers a large number of OTUs, shared with the root, that were not found in the other above-ground organs (see ^64^ and discussion therein). Further studies are needed to confirm ErM and DSE fungal distribution in aerial parts of *V. myrtillus*, but their occurrence in the floral parts opens intriguing questions on their possible vertical transmission via seeds, a phenomenon already described in forbs^65^.

In conclusion, we have described by metabarcoding the diversity of fungal assemblages associated with the endosphere of below-ground and above-ground organs of *V. myrtillus*. The results indicate niche differentiation in the *V. myrtillus* fungal microbiota, but they also revealed that some fungi so far considered as strict root symbionts can occupy different niches within the plant.

Several examples of fungi displaying dual life niches have been reported^66^. Some ErM fungi were already known to behave as dual saprotrophs/symbionts, with different root-interacting strategies according to the plant hosts^37^. Although further investigations are required, we showed here that ErM fungi may occupy a further ecological niche inside aerial plant parts. Promotion of host growth by ErM fungi has been mainly ascribed to the nutrient exchanges across the plant-fungus symbiotic interface formed around the intracellular hyphal coils. If these fungi play any role in promoting plant survival and growth in the aerial plant compartments, it is likely that other so far unknown mechanisms may take place.

## Methods

### Sampling site and description

The sampling site (45°50′40′′ N, 7°34′41′′ E, 2200 m a.s.l.; Supplementary Fig. S8) was a subalpine meadow, unused for 10 years, associated with the ICOS network (Integrated Carbon Observation System; station ID: IT-Tor) and managed by ARPA Valle d’Aosta (Regional Agency for the Environment Protection). In this site dominant taxa were different *Vaccinium* species (*V. myrtillus*, *V. gaultheroides*, *V. vitis-idaea*), *Rhododendron* sp., *Juniperus* sp., *Larix decidua*. Five clumps of soil with understorey vegetation were collected (Supplementary Fig. S8) and stored at 4 °C overnight. Soil was then washed away and roots of *V. myrtillus* were manually separated from roots of other plant species. The *V. myrtillus* roots were further washed (at least 2 h 30′ under running tap water) to remove any soil residues. Separate pools of roots, stems, leaves and flowers of plants from each clump were surface sterilized in NaClO 1% for 1 min and washed five times with sterile distilled H_2_O. We thus collected five pooled samples (n = 5, one from each clump) of the four different plant organs. From each pool, at least 4 subsamples of each organ were obtained and immediately stored at -80 °C. All the plant experiments were in compliance with relevant institutional, national, and international guidelines and legislation.

### DNA extraction, amplification and sequencing

The total DNA was extracted (NucleoSpin Plant II, Macherey–Nagel) from at least four subsamples of each organ. The ITS2 region was amplified by a two round PCR: (1) the full ITS region was amplified from the DNA extract with primers ITS1F (5′-CTTGGTCATTTAGAGGAAGTAA-3′) and the ITS4 (5′-TCCTCCGCTTATTGATATGC-3′); (2) the ITS2 region was amplified, by a semi-nested approach, from the product of the first amplification (1:10 v/v dilution) with primers ITS9fngs (5′-GAACGCAGCRAAIIGYGA-3′) and ITS4ngs (5′-TCCTCCGCTTATTGATATGC-3′), both added to Illumina overhang adapter sequences: forward overhang 5′-TCGTCGGCAGCGTCAGATGTGTATAAGAGACAG-[locus specific target primer]-3′, reverse overhang: 5′-GTCTCGTGGGCTCGGAGATGTGTATAAGAGACAG-[locus specific target primer]-3′. The obtained PCR products were checked on 1% agarose gel. The products obtained from different subsamples of the same sample were pooled together before being purified (Wizard SV Gel and PCR CleanUp System, Promega), quantified with Qubit 2.0 (Thermo Fisher Scientific, Waltham, MA, USA) and sent for Illumina MiSeq 2 × 300 bp sequencing to IGA Technology Services Srl (Udine, Italy).

#### Bioinformatics

Sequencing adapters and primers were removed and then paired‐end reads from each sample were merged with Pear v.0.9.2^67^ using a quality score threshold set at 28 and a minimum length after trimming set at 200 bp. The assembled reads were then processed using the Quantitative Insights into Microbial Ecology (Qiime) v.1.9.1 software package^68^. Sequence processing and sample assignment were performed with a minimum sequence length cut-off of 200 bp and a Phred quality score of 28, calculated over a sliding window of 50 bp. Chimeric sequences were removed performing a de novo detection using UCHIME^69^. OTUs were obtained using VSEARCH^70^ at 97% similarity, and taxonomically assigned using the Full UNITE + INSD dataset for Fungi Version 10.05.2021 (UNITE Community 2021^71^; Supplementary Table S1). BLAST algorithm^72^ was used as taxonomy assignment method, with 1e^-5^ e-value as threshold (Supplementary Table S2). A more precise assignment of the OTUs in the *Hyaloscypha* and *Oidiodendron* genera was supported by the phylogenetic analysis performed according to published methods^30^. Briefly, Bayesian analysis and Maximum Likelihood approaches were used for phylogenetic tree construction using MrBayes v. 3.2.6^73^ and MEGAX^74^, after alignment with ClustalW with default parameters. Reference sequences from public databases are listed in Supplementary Table S3^30,34,75^.

The statistical and visual analyses on the OTUs have been performed by the Marker Data Profiling tool of MicrobiomeAnalist^76^. OTUs for which at least 10% of their counts in the different samples contained at least 10 reads have been retained. OTUs with a standard deviation lower than 5% throughout the experimental conditions were discarded. Data were rarefied to the sample with the lowest sequencing depth. Data have not been scaled a priori.

## Supplementary Information

Supplementary Information 1.

Supplementary Information 2.

Supplementary Information 3.

## Data Availability

The raw sequences from the metabarcoding experiment have been deposited with the BioProject ID PRJNA769432 (https://www.ncbi.nlm.nih.gov/bioproject/?term=PRJNA769432). The data-sets generated and analyzed during the current study are included in this published article (and its Supplementary Information files).
