# Supplementary material for: Metabarcoding of fungal assemblages in Vaccinium myrtillus endosphere suggests colonization of above-ground organs by some ericoid mycorrhizal and DSE fungi
Source: Sci Rep. 2022 Jun 30;12:11013. doi: 10.1038/s41598-022-15154-1 (PMC9246922; doi:10.1038/s41598-022-15154-1)
Supplement: Supplementary file 1 — Supplementary Information 1. [file 41598_2022_15154_MOESM1_ESM.pdf]

Metabarcoding of fungal assemblages in *Vaccinium myrtillus* endosphere suggests colonization of above-ground organs by some ericoid mycorrhizal and DSE fungi

Authors:

Stefania Daghino[1], Elena Martino[2], Samuele Voyron[1,2], Silvia Perotto[2,*]

[1] Institute for Sustainable Plant Protection, CNR, Strada delle Cacce 73, 10135 Torino and V. le Mattioli 25, 10125 Torino, Italy.

[2] Department of Life Science and Systems Biology, University of Torino, V. le Mattioli 25, 10125, Torino, Italy.

Figure S1. Alpha-diversity of the fungal assemblages associated with the different plant organs. The alpha-diversity associated with each organ has been calculated according to a) Chao1 and b) Shannon diversity metrics with following parameters: data input: filtered, experimental factor: organ, taxonomic level: feature, statistical method: Mann-Whitney/Kruskal-Wallis.

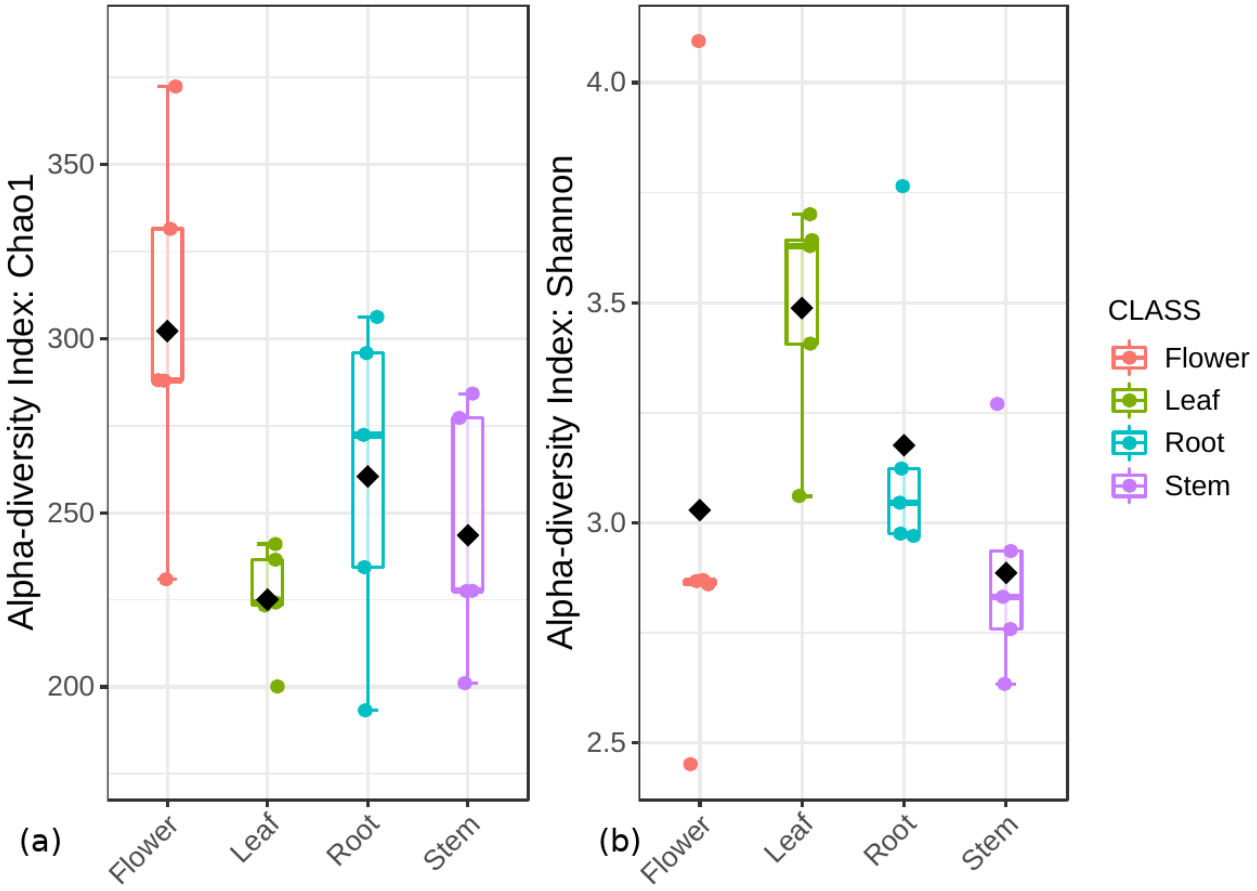

Figure S2. Description of the fungal diversity associated with different plant organs. (a) relative abundance of phyla, (b) phyla with statistically different abundance among the organs (Pairwise Wilcoxon rank sum test/Kruskal-Wallis or ANOVA with BH-adjusted p-val<0.05).

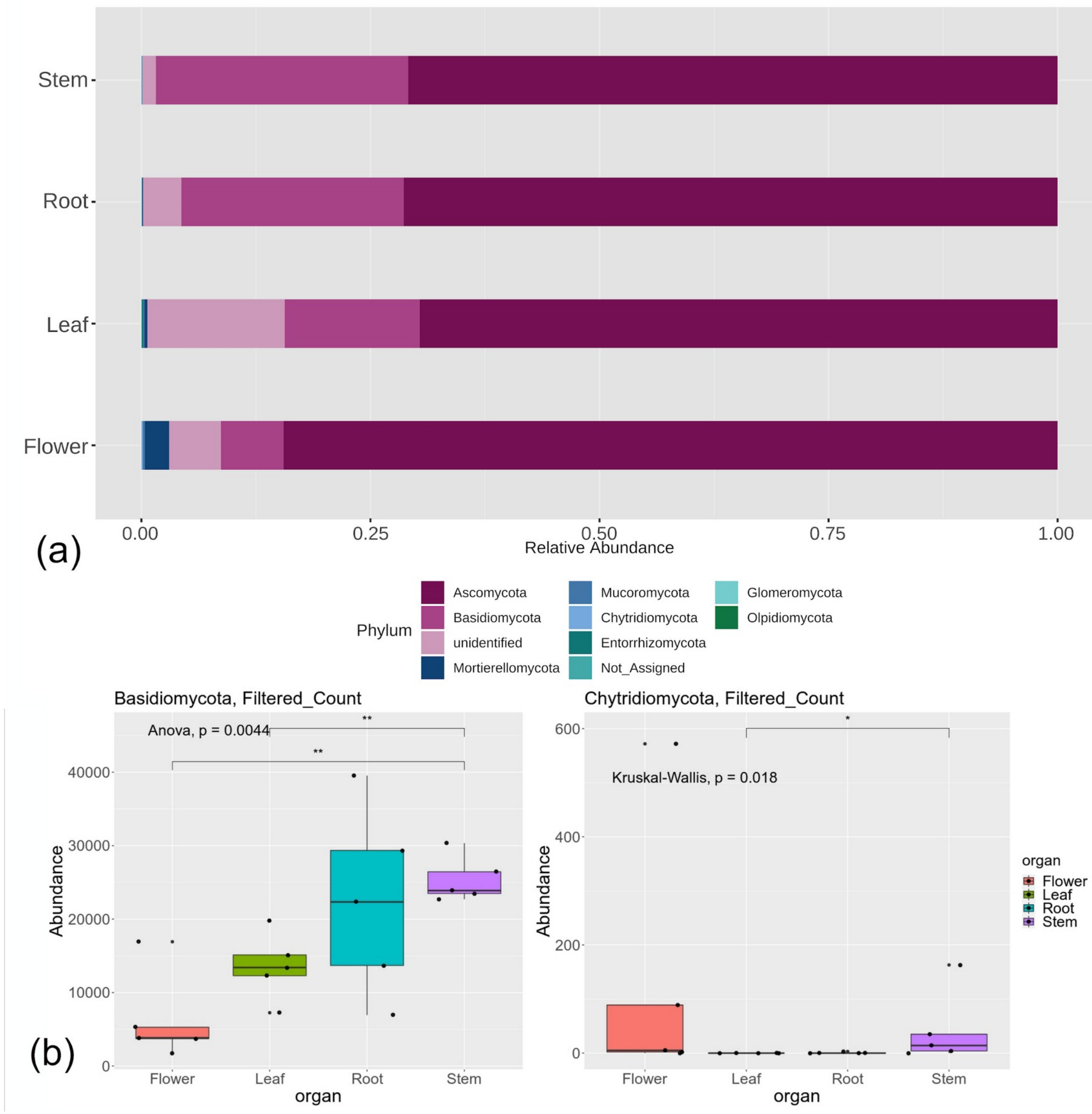

Figure S3. Fungal classes associated with different plant organs: classes with statistically different abundance among the organs (Pairwise Wilcoxon rank sum test/Kruskal-Wallis with BH-adjusted p-val<0.05).

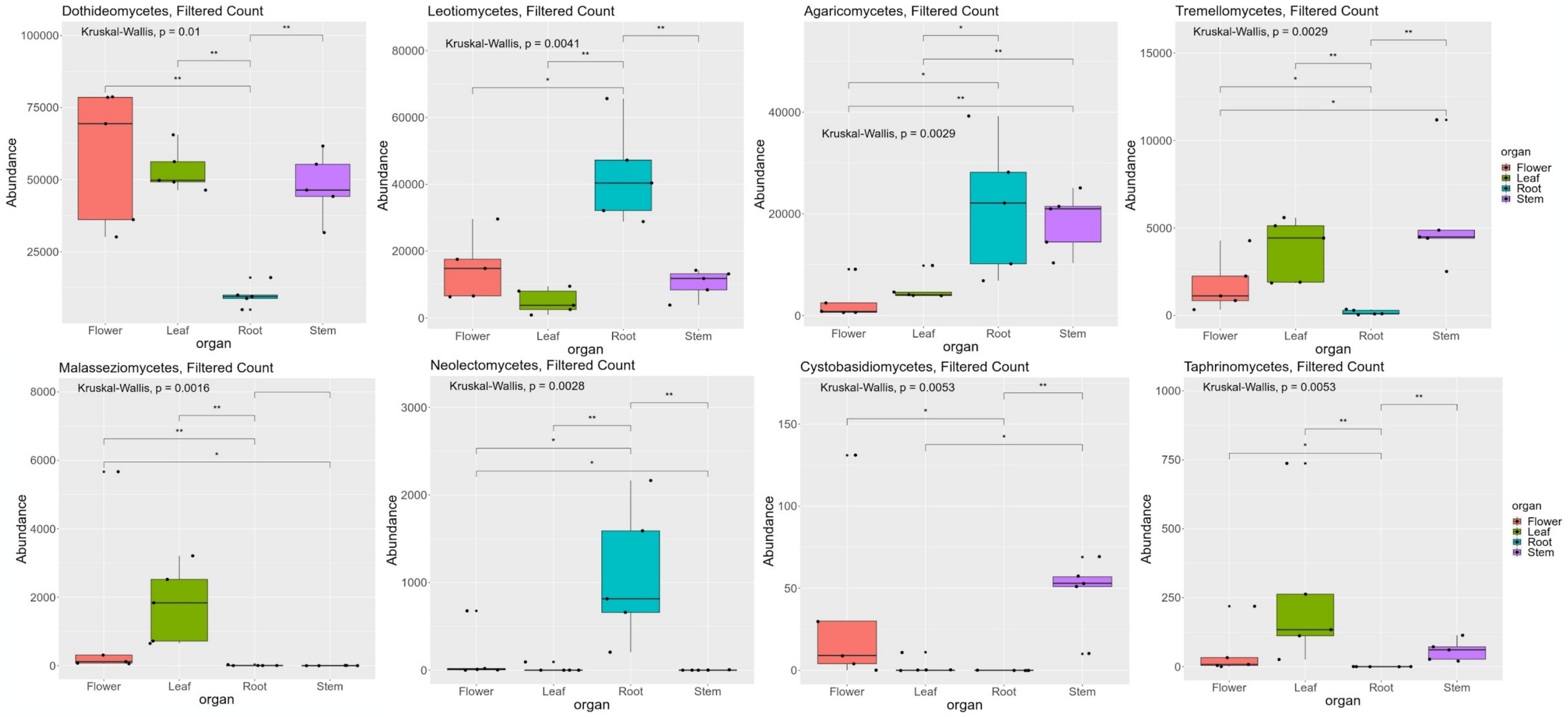

Figure S4. Description of the fungal diversity associated with different plant organs: relative abundance of fungal genera.

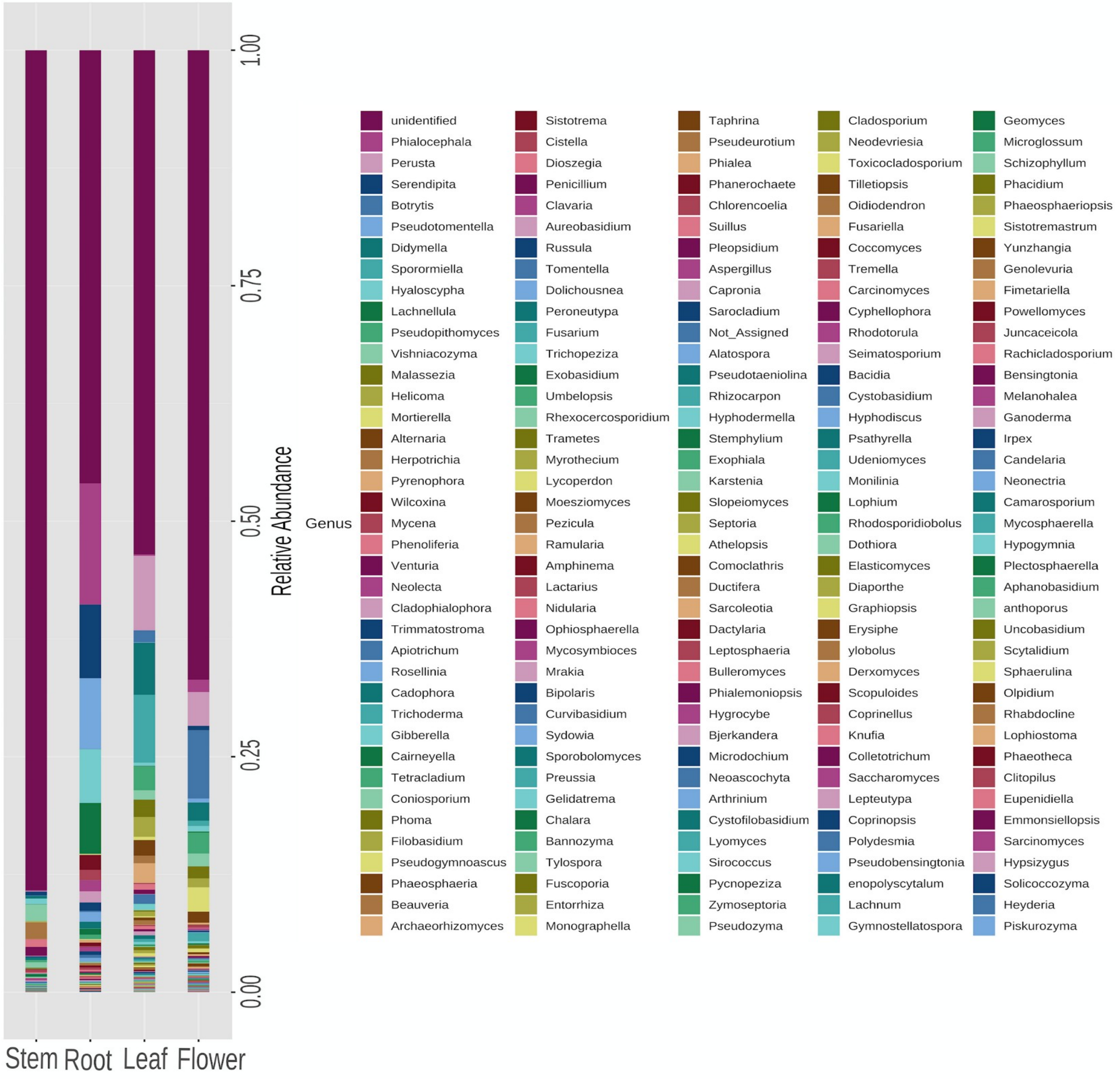

Figure S5. Phylogenetic analysis of the OTUs attributed to the *Hyaloscypha* spp. ITS2 sequences from the OTUs attributed to *Hyaloscypha* spp. by matching with the Unite database, have been aligned. The Bayesian consensus tree is shown with posterior probabilities (pp). Bootstrap support (bs) for maximum likelihood analyses is given if >50%. These values are reported close to branches separated by hyphen (pp-bs). Taxa names are maintained as in GenBank (accession numbers included). The OTUs found in this paper are indicated by their respective ID number.

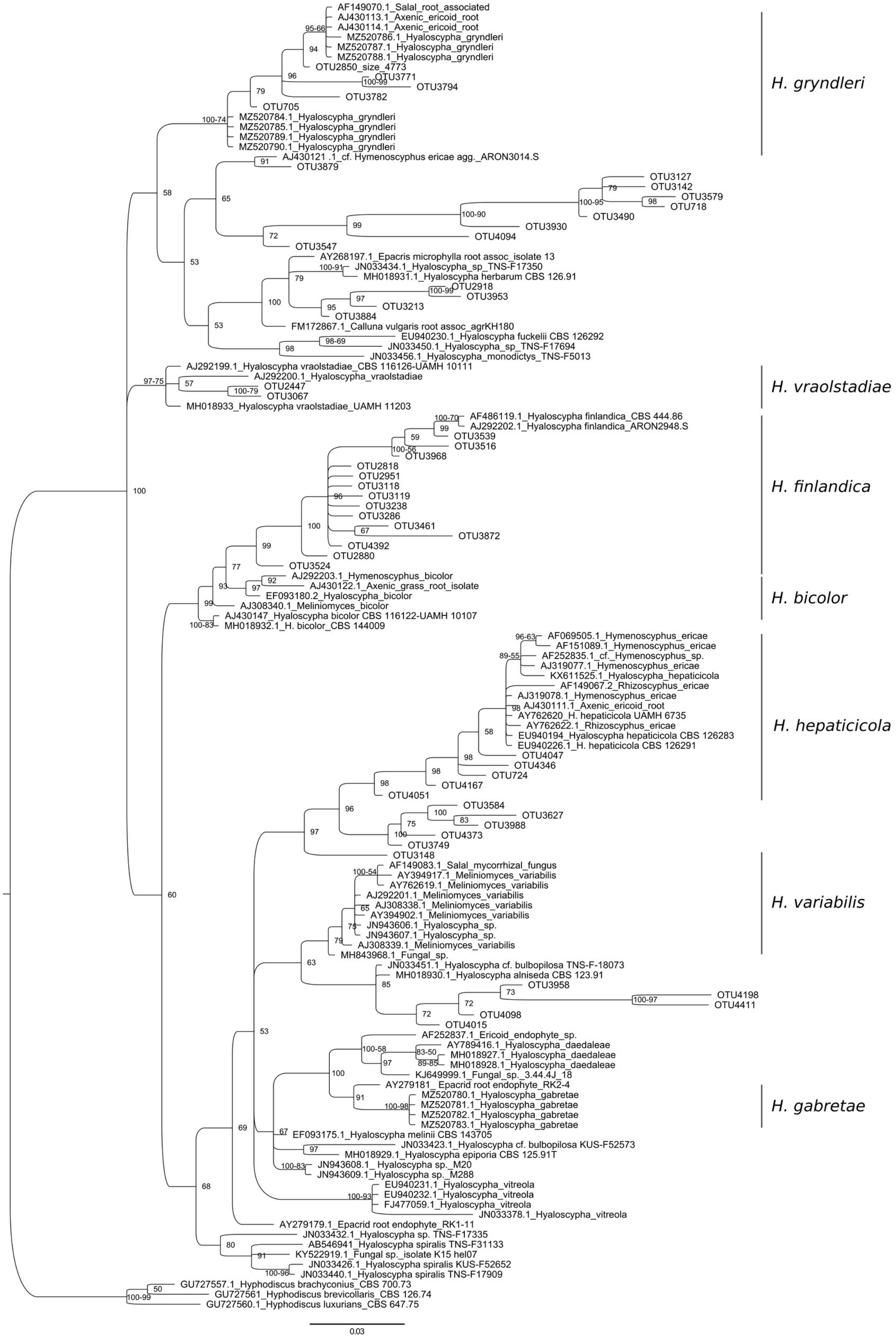

Figure S6. Phylogenetic analysis of the OTUs attributed to *Oidiodendron* spp. ITS2 sequences from the OTUs attributed to the *Oidiodendron* genus by matching with the Unite database have been aligned. The Bayesian consensus tree is shown with posterior probabilities (pp). Bootstrap support (bs) for maximum likelihood analyses is given if >50%. These values are reported close to branches separated by hyphen (pp-bs). Taxa names are maintained as in GenBank (accession numbers included). The OTUs found in this paper are indicated by their respective ID number.

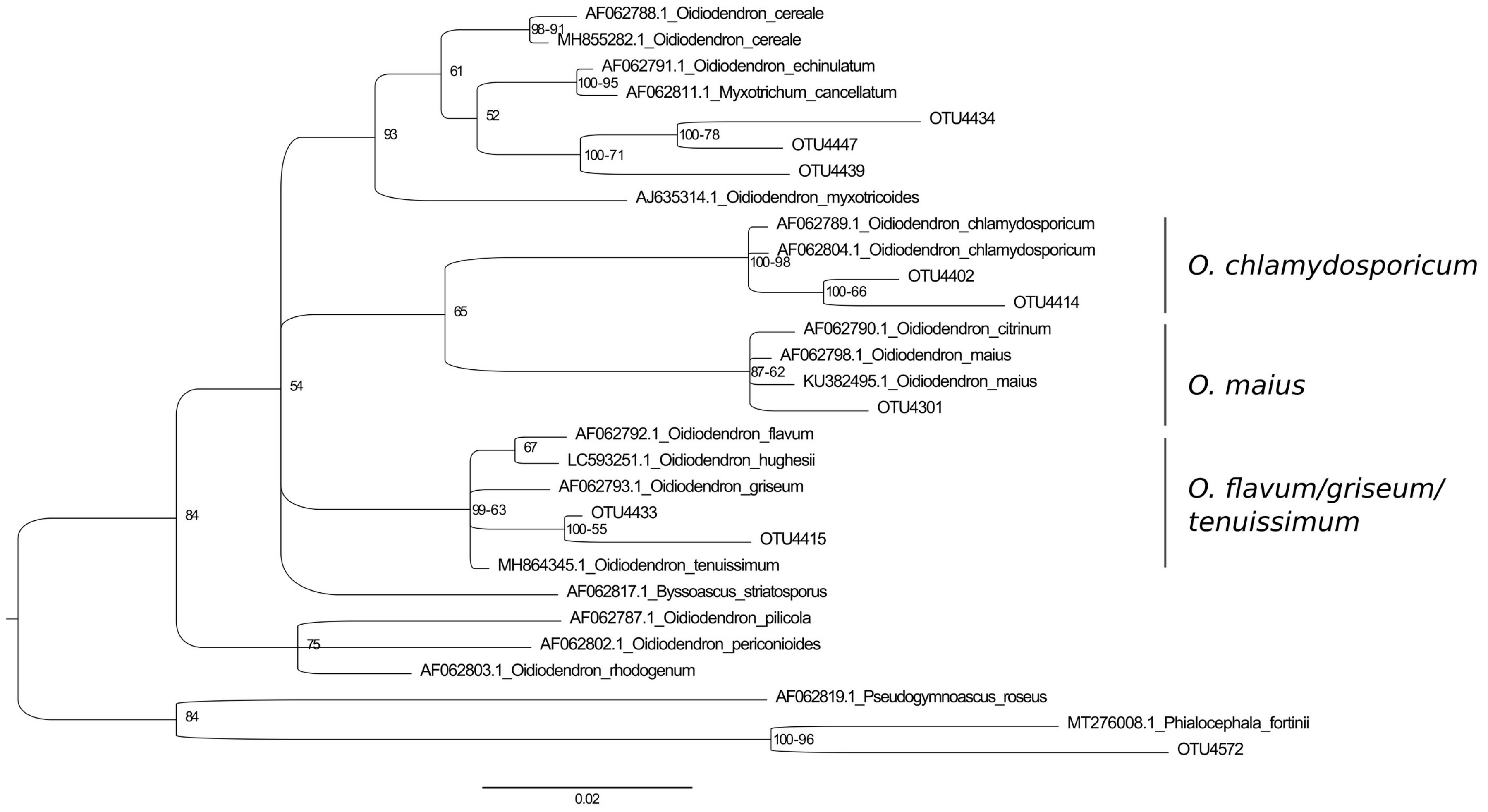

Figure S7. Hub taxa in each organ defined by Linear discriminant analysis Effect Size (LefSe). a) classes, b) orders and c) genera that best characterize each organ (LDA score>3 and Kruskal–Wallis p-val<0.05) are ranked in decreasing order by their LDA scores (x axis). The mini-heatmap to the right of the plot indicates whether the taxa are more (red) or less (blue) represented in each organ. (d) Principal Component Analysis showing the distances between the samples.

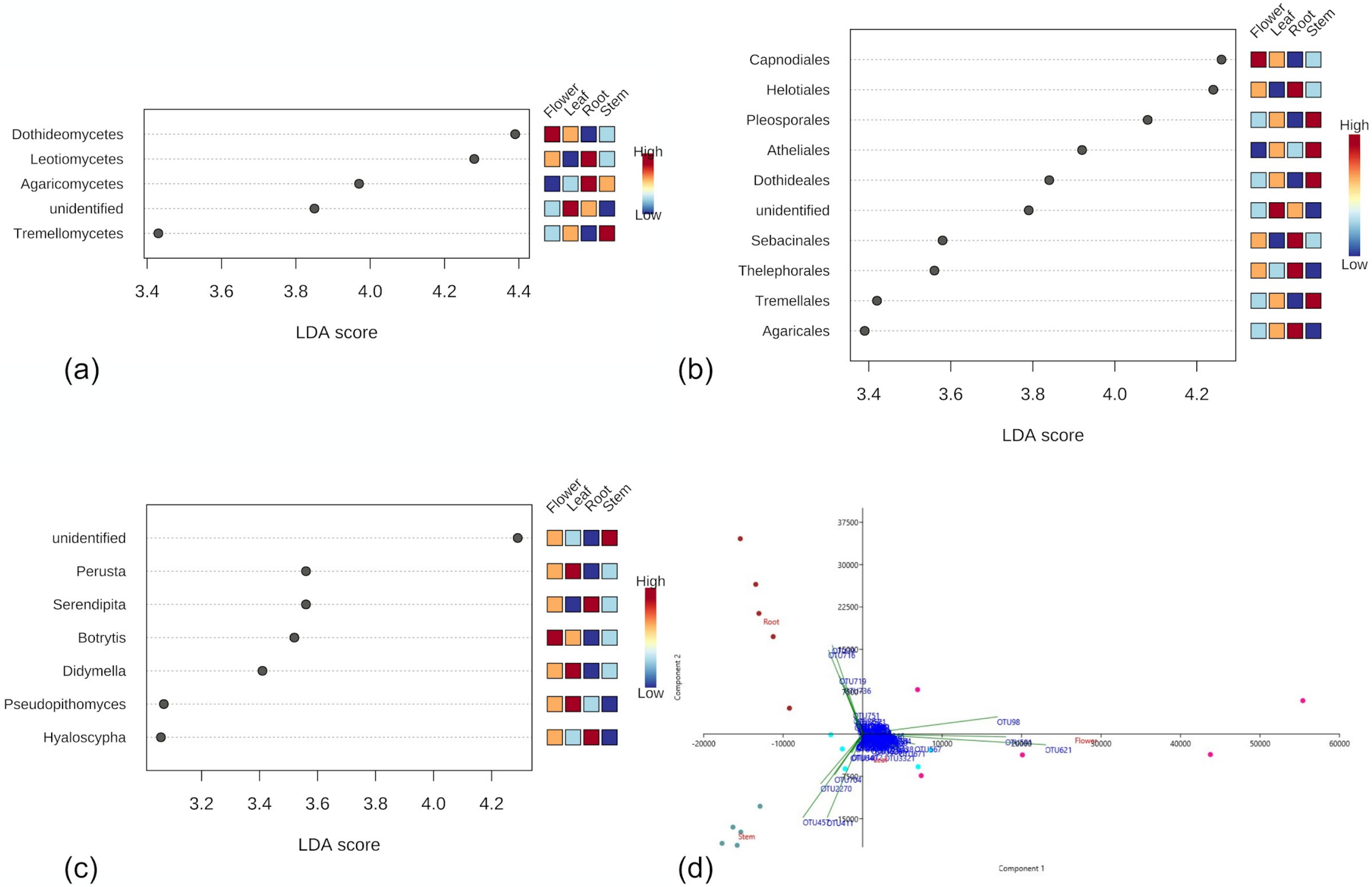

Figure S8. (A) Geographic localization of the sampling site (map modified from OpenStreetMap, licensed under the Open Data Commons Open Database License (ODbL) by the OpenStreetMap Foundation (OSMF). - Copyright and licence: https://www.openstreetmap.org/copyright/en), with the approximate position where the 5 samples have been collected; (B) panoramic view of the sampling site; (C) pic of a single clump sample from which the plants have been pooled (see method section).

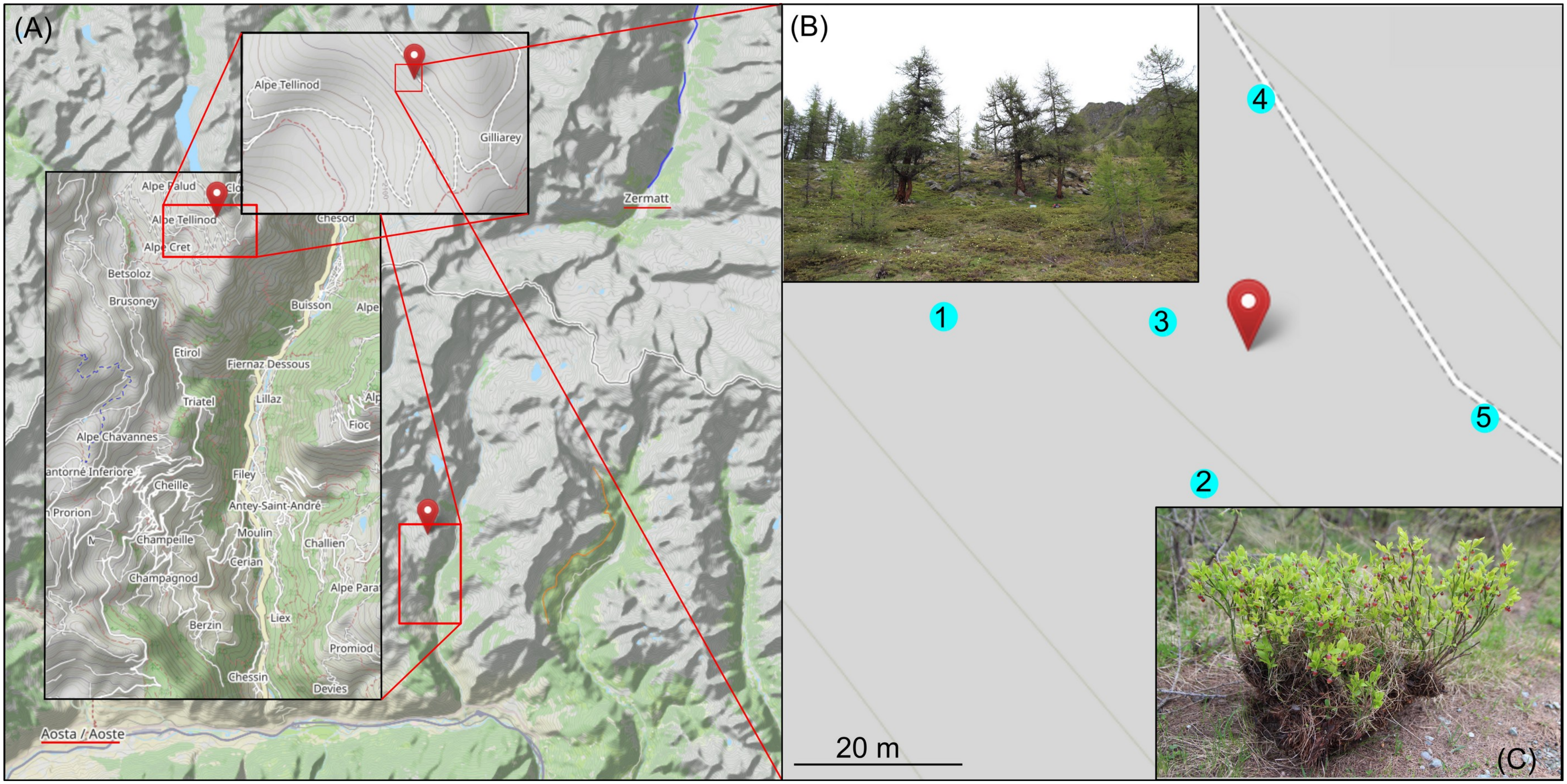

Table S3. GenBank accession numbers and taxa used in the phylogenetic analysis. References: 30, 34, 75.

Accession number	Taxon description
AB546941	*Hyaloscypha spiralis*
AF062787.1	*Oidiodendron pilicola*
AF062788.1	*Oidiodendron cereale*
AF062789.1	*Oidiodendron chlamydosporicum*
AF062790.1	*Oidiodendron citrinum*
AF062791.1	*Oidiodendron echinulatum*
AF062792.1	*Oidiodendron flavum*
AF062793.1	*Oidiodendron griseum*
AF062798.1	*Oidiodendron maius*
AF062802.1	*Oidiodendron periconioides*
AF062803.1	*Oidiodendron rhodogenum*
AF062804.1	*Oidiodendron chlamydosporicum*
AF062811.1	*Myxotrichum cancellatum*
AF062817.1	*Byssoascus striatosporus*
AF062819.1	*Pseudogymnoascus roseus*
AF069505.1	*Hymenoscyphus ericae isolate 101*
AF149067.2	*Rhizoscyphus ericae*
AF149070	Salal root associated fungus
AF149083.1	Salal mycorrhizal fungus
AF151089.1	*Hymenoscyphus ericae*
AF252835.1	cf. Hymenoscyphus sp. GU27
AF252837	Ericoid endophyte
AF486119	*Hyaloscypha finlandica*
AJ292199	*Hyaloscypha vraolstadiae*
AJ292200.1	*Meliniomyces vraolstadiae*
AJ292201.1	*Meliniomyces variabilis*
AJ292202	*Hyaloscypha finlandica*
AJ292203.1	*Hymenoscyphus bicolor*
AJ308338.1	*Meliniomyces variabilis*
AJ308339.1	*Meliniomyces variabilis*
AJ308340.1	*Meliniomyces bicolor*
AJ319077.1	*Hymenoscyphus ericae*
AJ319078.1	*Hymenoscyphus ericae*
AJ430111.1	Axenic ericoid root isolate
AJ430113	Axenic ericoid root isolate
AJ430114	Axenic ericoid root isolate
AJ430121	cf . Hymenoscyphus ericae agg.
AJ430122.1	Axenic grass root isolate
AJ430147	*Hyaloscypha bicolor*
AJ635314.1	*Oidiodendron myxotrichoides*
AY268197	*Epacris microphylla root assoc. fungus*

AY279179	Epacrid root endophyte
AY279181	Epacrid root endophyte
AY394902.1	*Meliniomyces variabilis*
AY394917.1	*Meliniomyces variabilis*
AY762619	*Hyaloscypha variabilis*
AY762620	*Hyaloscypha hepaticicola*
AY762622.1	*Rhizoscyphus ericae*
AY789416	*Hyaloscypha daedaleae*
EF093175	*Hyaloscypha melinii*
EF093180.2	*Hyaloscypha bicolor*
EU940194	*Hyaloscypha hepaticicola*
EU940226	*Hyaloscypha hepaticicola*
EU940230	*Hyaloscypha fuckelii*
EU940231	*Hyaloscypha vitreola*
EU940232	*Hyaloscypha vitreola*
FJ477059	*Hyaloscypha vitreola*
FM172867	*Calluna vulgaris root assoc. fungus*
GU727557	*Hyphodiscus brachyconius*
GU727560	*Hyphodiscus luxurians*
GU727561	*Hyphodiscus brevicollaris*
JN033378	*Hyaloscypha vitreola*
JN033423	*Hyaloscypha cf. bulbopilosa*
JN033426	*Hyaloscypha spiralis*
JN033432	*Hyaloscypha sp.*
JN033434	*Hyaloscypha sp.*
JN033440	*Hyaloscypha spiralis*
JN033450	*Hyaloscypha sp.*
JN033451	*Hyaloscypha cf. bulbopilosa*
JN033456	*Hyaloscypha monodictys*
JN943606	*Hyaloscypha sp.*
JN943607	*Hyaloscypha sp.*
JN943608	*Hyaloscypha sp.*
JN943609	*Hyaloscypha sp.*
KJ649999	Fungal sp.
KU382495.1	*Oidiodendron maius*
KX611525.1	*Hyaloscypha hepaticicola*
KY522919	Fungal sp. ericoid mycorrhizal isolate
LC593251.1	*Oidiodendron hughesii*
MH018927	*Hyaloscypha daedaleae*
MH018928	*Hyaloscypha daedaleae*
MH018929	*Hyaloscypha epiporia*
MH018930	*Hyaloscypha alniseda*
MH018931	*Hyaloscypha herbarum*
MH018932	*Hyaloscypha bicolor*

MH018933	*Hyaloscypha vraolstadiae*
MH843968	Fungal sp. root isolate
MH855282.1	*Oidiodendron cereale*
MH864345.1	*Oidiodendron tenuissimum*
MT276008.1	*Phialocephala fortinii*
MZ520780	*Hyaloscypha gabretae*
MZ520781	*Hyaloscypha gabretae*
MZ520782	*Hyaloscypha gabretae*
MZ520783	*Hyaloscypha gabretae*
MZ520784	*Hyaloscypha gryndleri*
MZ520785	*Hyaloscypha gryndleri*
MZ520786	*Hyaloscypha gryndleri*
MZ520787	*Hyaloscypha gryndleri*
MZ520788	*Hyaloscypha gryndleri*
MZ520789	*Hyaloscypha gryndleri*
MZ520790	*Hyaloscypha gryndleri*
